# Future treatment options for facial nerve palsy: a review on electrical stimulation devices for the orbicularis oculi muscle

**DOI:** 10.1007/s10072-023-07226-5

**Published:** 2023-12-20

**Authors:** Elena Scherrer, Karla Chaloupka

**Affiliations:** Department of Ophthalmology, University Hospital Zurich, University of Zurich, Zurich, Switzerland

**Keywords:** Electrical stimulation, Facial reanimation, Orbicularis oculi muscle, Facial nerve palsy

## Abstract

Facial nerve palsy can cause diminished eyelid closure (lagophthalmos). This occurs due to functional deficits of the orbicularis oculi muscle, potentially leading to sight-threatening complications due to corneal exposure. Current management options range from frequent lubrication with eye drops, to the use of moisture chambers and surgery. However, achieving functional restoration may not always be possible. Recent efforts have been directed towards the support of orbicularis oculi muscle function through electrical stimulation. Electrical stimulation of the orbicularis oculi muscle has been demonstrated as feasible in human subjects. This article offers a comprehensive review of electrical stimulation parameters necessary to achieve full functionality and a natural-looking eye blink in human subjects. At present, readily available portable electrical stimulation devices remain unavailable. This review lays the foundation for advancing knowledge from laboratory research to clinical practice, with the ultimate objective of developing a portable electrical stimulation device. Further research is essential to enhance our understanding of electrical stimulation, establish safety standards, determine optimal current settings, and investigate potential side effects.

## Introduction

Facial palsy is frequently seen with a range of 17 to 35 cases per 100,000 [1, 2]. The facial nerve contains the mixed parasympathetic innervation of the lacrimal gland for reflex tearing and the efferent fibers for the ocular orbicularis muscle [2]. The palpebral part of the ocular orbicularis muscle is involved in gentle eyelid closure and is an essential part of the blink reflex, and the tear pump system transporting tears into the lacrimal sac [3, 4]. The orbital part of the orbicularis muscle is involved in the forced eyelid closure [3]. From an ophthalmological perspective, the weakening of or loss of function of the facial nerve leads to impaired eyelid closure, resulting in lagophthalmos, which can subsequently cause corneal damage [1]. Corneal exposure keratopathy can be sight-threatening and have a huge impact on quality of life [1, 5]. The risk of developing an exposure keratopathy with sight-threatening corneal ulceration is multiplied with a concomitant impaired trigeminal function.

Reasons for a facial palsy are numerous [2]. Common causes of facial palsy include Bell’s palsy, benign and malignant tumors including perineural invasion, iatrogenic facial nerve injuries, trauma, Varicella zoster–associated facial palsy, Lyme disease, autoimmune disease, congenital and hereditary causes, or stroke [6, 7].

To address ophthalmic complications arising from facial nerve palsy, the current treatment options encompass intensive topical lubrication, utilization of moisture chambers, and botulinum toxin injections targeting the levator muscle [1, 8]. Common surgical treatment options include upper lid gold-weight implants, lateral and medial canthal suspension, or, in rare cases, tarsorrhaphy. Several alternative surgical options are available; however, patients frequently experience enduring damage or unsatisfactory cosmetic results. Consequently, innovative treatment modalities for ophthalmic complications are crucial in improving the quality of life for patients [1, 9].

Recently, a novel treatment approach for ophthalmic complications resulting from facial nerve palsy is under evaluation. It has been demonstrated that eyelid closure can be achieved through electrical stimulation of the ocular orbicular muscle [10–15]. During the initial phase of facial palsy, the musculature retains its integrity. However, insufficient stimulation can lead to muscular atrophy. There is some evidence suggesting that early electrical muscle stimulation might help prevent such atrophy [16–18]. Therefore, employing electrical stimulation of the orbicularis oculi may hold potential for preventing common ophthalmic complications associated with facial nerve palsy.

The majority of experiments involving electrical stimulation were conducted on animal models [19–25]. Nevertheless, certain research groups have investigated the characteristics of electrical stimulation of the orbicularis oculi in human subjects [10–15, 26–28]. Subsequent trials have focused on achieving a natural-looking eye blink in the paralyzed orbicularis oculi muscle through synchronized electrical stimulation, with the non-affected healthy side [11, 26–29]. These efforts aim to develop a portable prosthesis device for patients with facial nerve palsy.

The objective of this review is to provide a comprehensive summary and comparison of various electrical stimulation parameters for the orbicularis oculi muscle that would be required for a portable electrical stimulation device in humans, enabling a natural and symmetrical eye closure. To the best of our knowledge, no such electrical stimulation device for the orbicularis oculi muscle in human clinical practice currently exists. Therefore, the findings from this review could serve as a starting point for the development of a clinical model.

## Method of literature search

The review was conducted based on an online literature search in the MEDLINE database (https://www.nlm.nih.gov/bsd/medline.html) via PubMed (https://pubmed.ncbi.nlm.nih.gov/), and the Cochrane Library (https://www.cochranelibrary.com) using the following key terms: “facial palsy,” “facial nerve,” “facial paralysis,” “electrical stimulation,” “orbicularis oculi.” Furthermore, the reference list of the obtained papers was screened for suitable publications. During the screening process, we included all publications irrespective of their language or publication date, up to January 2022. We thoroughly reviewed the acquired literature and selected or excluded articles based on their relevance to our research aim of investigating electrical stimulation of the orbicularis oculi muscle in human subjects.

## How to achieve an eye blink in facial nerve palsy using electrical stimulation with a closed-loop system device in human subjects

To achieve a natural-looking eye blink, synchronous eye closure with the healthy side should be strived for. Human perception typically does not detect facial muscle movement asymmetry of less than 33 ms [15, 30, 31]. For the design of a device that stimulates the paretic orbicularis oculi muscle in synchronization with the healthy side, early detection of muscle activity and processing of information from the healthy eye’s muscle activity are crucial.

In a normal eye blink, the time delay from orbicularis oculi activation to the onset of movement is approximately 10–12 ms [32]. Early detection of muscle activity is necessary to initiate a rapid triggering stimulus to the impaired eyelid. The time from the onset of electrical stimulation of the orbicularis oculi to muscle movement has been reported to average around 10 ms [15]. This process of stimulus application based on healthy muscle activity is known as a closed-loop electrical stimulation system [10, 11, 14, 15, 23, 26–28]. In a closed-loop electrical stimulation system, the time interval between stimulus detection on the healthy eye and induction of lid movement on the paretic side should not exceed 33 ms.

Physiologically, during a normal eye blink, the levator and the orbicularis oculi muscles have antagonistic functions. The relaxation of the levator muscle precedes the activation of the orbicularis oculi. Much of the research focuses on detecting activity in the orbicularis oculi muscle of the healthy eye to trigger an electrical stimulation signal to the paretic orbicularis oculi muscle [11, 14, 15, 23, 26–28]. However, there are also discussions about measuring the cessation of the levator muscle on the affected side, which is innervated by the third cranial nerve [28, 33]. Nonetheless, this approach to electrical stimulation poses challenges due to potential interference from stimulation artifacts in the orbicularis oculi muscle and levator activity [28]. Consequently, a less error-prone approach may involve basing the stimulation of the affected orbicularis muscle on the muscle activity observed on the contralateral healthy side.

## How can electrical stimulation of the orbicularis oculi muscle induce eye closure in human subjects?

To implement electrical stimulation in the paretic orbicularis oculi muscle in the future, a thorough investigation of its electrical stimulation characteristics in human subjects is essential. The electrical stimulation should be capable of inducing lid closure without causing pain or other adverse effects. Therefore, understanding suitable electrical current settings and identifying the ideal application site for the electrical stimulus is of paramount importance.

### Electrical stimulation settings needed to induce sufficient eye closure of the orbicularis oculi muscle

Complete lid closure, both during the day and at night, is vital for preventing corneal exposure keratopathies. Additionally, the protective Bell phenomenon, which safeguards the cornea while the lid is closed, occurs only during complete or intended lid closure. Studies have demonstrated that electrical stimulation of the orbicularis oculi muscle can induce eye closure in human subjects [11–15, 21]. In animal models, lid closure elicited by electrical stimulation shows an approximately linear response, with greater electrical stimuli resulting in greater palpebral fissure closure [19–21]. Similarly, an increase in pulse width leads to increased lid closure. However, once the electrical pulse width exceeds a certain limit, a decrease in lid closure is observed. This effect might be attributed to long biphasic pulses, which activate different muscle groups with each pulse phase, leading to interference between stimuli [19].

Other studies have found that a pulse train of several pulses leads to more natural-looking and functional results compared to a single pulse stimulation of the orbicularis oculi [10, 19, 26]. Various single pulse and pulse train stimulation patterns have been tested on human subjects, as listed in Table 1. Pulse train stimulation induces contraction at lower pulse amplitude ranges and has been shown to reduce electric current by up to 40% compared to single pulse stimulation [15, 19, 26]. In animal models, Sachs et al. demonstrated that the effect of orbicularis oculi stimulation reaches its plateau at 10 pulses per pulse train [19]. The observed phenomenon of better lid closure in pulse train stimulation than in single pulse stimulation is believed to be due to wave summation and tetanic contraction [10].

**Table 1:** Settings of electrical stimulation for the orbicularis oculi muscle in different human studies

Authors	Publication year	Patient characteristics, number of participants (N)	Pulse train	Pulse width	Pulse amplitude/voltage	Frequency	Electrodes and location	Eye closure	Side effects
[Reference]				in ms					
McDonnall D, Guillory KS, Gossman MD [14]	2009	Patients with facial nerve palsy, *N* = 6	Biphasic pulse trains	5	0.4 ± 0.1 V	60 ms pulse trains at 50 Hz	Implantation of 4 platinum microwires 1 mm into the upper eye lid, 2 mm above lid margin	Incomplete	• Not systematically reported • 4/5 patients with significant motor response, prior to reaching highest discomfort level
			Pulse train with interferential stimulation			Interferential stimulation frequency of 30 Hz	Surface electrodes over orbicularis oculi muscle	Incomplete	• Not systematically reported • 2/3 patients with complete functional blink below maximal discomfort level
Frigerio A, Cavallari P [11]	2012	Healthy volunteers, *N* = 10	10 pulses	0.8	26 V	Two initial pulses (with dynamic pulse range) 250–300 Hz, then carrier frequency 75–200 Hz	Surface electrodes along zygomatic branch of the facial nerve	Complete	• Not systematically reported • Painful Stimulation at frequencies lower than 100 Hz
Marcelli E, Cavallari P, Frigerio A, et al. [10]	2013	Healthy volunteer, *N* = 1	1 pulse	2	8 mA		Surface electrodes on temporal canthus of the eye	Complete	• Not systematically reported • Painful, longer than normal reflex eye blink in the contralateral healthy eye
			10 pulses	2.0	3.5 mA	200 Hz			
			10 pulses	1.0	3.5 mA	200 Hz			
			10 pulses	0.5	4.0 mA	200–400 Hz			
Frigerio A, Heaton JT, Cavallari P, et al. [15]	2015	Patients with facial nerve palsy, *N* = 40	Pulse trains with shorter intervals between the first two pulses	0.4–1	6.2–7.8 mA (mean 7.2 mA); identical voltage for the whole pulse train	100–150 Hz	Surface electrodes along zygomatic branch of the facial nerve	Complete in 55% (22/40)	• 2/40 (5%) patients aborted trial at 8 mA because of discomfort • 16/22 patients (which achieved full eye closure) reported pain • Synkinesis at 12-week follow-up reported (not directly related to electrical stimulation)
Ilves M, Lylykangas J, Rantanen V, et al. [12]	2019	Healthy volunteers, *N* = 24	Pulse train (duration 80 ms)	0.4	2.5–5 mA (mean 3.6 mA)	250 Hz	Surface electrodes above orbicularis oculi muscle	Complete in 22/24 (91.7%) patients	Pain scale from 1 (no pain) to 9 (severe pain): • Mean pain rating for current threshold to elicit eye twitch: 1.8 (SD 1.5, *n* = 16) • Mean pain rating for current threshold to elicit eye blink: 3 (SD 1.7, *n* = 20);
Mäkelä E, Venesvirta H, Ilves M, et al. [13]	2019	Patients with facial nerve palsy, *N* = 24	Pulse train (duration 80 ms)	0.4	2.3 mA ± 0.9 (SD) and 4.7 mA ± 2.2 (SD)	250 Hz	Surface electrodes above orbicularis oculi muscle	Incomplete	Pain scale from 1 (no pain) to 9 (severe pain): • Mean pain rating 4.3 (± 2.6 SD)

In contrast to physiological eyelid closure, single pulse stimulation can cause painful and prolonged eyelid closure as well as induce reflex blinking in the contralateral eye [10]. Frigerio et al. also suggested that introducing dynamic frequencies in pulse train stimulation may be beneficial, particularly by shortening the interval between the first two pulses. They observed that dynamic frequencies resulted in a faster peak acceleration of the eyelid compared to using a constant frequency between all the pulses, leading to a 15% reduction in stimulation, which they attributed to the dynamic sensitivity of motoneurons [11, 15].

### Electrode placement for orbicularis oculi muscle stimulation

Electrode placement for electrical stimulation of the orbicularis oculi muscle in human subjects has been previously described. The most commonly utilized locations for electrode placement are along the zygomatic branch of the facial nerve at the temporal orbit rim [10, 11, 15], or above the orbicularis oculi muscle [12–14]. In both cases, complete eyelid closure was observed [10–12, 15].

In a study by McDonnall et al. four microwires were implanted 1 mm into the upper eyelid to stimulate the orbicularis muscle; however, no eyelid closure was achieved [14]. Due to the thin nature of the orbicularis oculi muscle in humans, implanting electrodes into the muscle belly can lead to the possibility of missing the muscle. Moreover, bleeding or edema resulting from electrode implantation could impede electrical stimulation. A separate study revealed a positive correlation between BMI and electric stimulation amplitude levels for forehead and cheek movements. The authors attributed this correlation to the fact that people with higher BMI tend to have more fat tissue, requiring higher currents to elicit muscle movement [12]. However, no such study has been conducted for the orbicularis oculi muscle. To avoid complications such as bleeding or edema, the use of surface electrodes is a suitable alternative, which do not require surgery for implantation.

So far, different electrode placement settings for direct orbicularis oculi stimulation have only been compared in animal models. Zhang et al. [21] conducted a study in rabbits to investigate the distribution of horizontal and vertical electrode arrays along the orbicularis oculi muscle and analyzed the electrical stimulation characteristics. They noted that the electric distribution field of the orbicularis oculi muscle has an oval shape, with a faster current conduction along the muscle fibers in the horizontal array compared to across muscle fibers in the vertical electrode array [21]. Therefore, to efficiently stimulate the orbicularis oculi with minimal current and maximal energy efficiency, the electrode placement should consider these differences in current distribution [21].

In another study, Somia et al. [22] tested lid closure in dogs by stimulating in a single electrical field and multiple-channel stimulation. For single-field electrical stimulation, they inserted two electrodes into the orbicularis oculi muscle, creating a horizontal electrical field along the orbicularis oculi fibers. In multiple-channel stimulation, they inserted four electrodes in the upper and four in the lower lid, thus creating four separate electrical fields along the orbicularis oculi muscle fibers [22]. They reported a significant reduction in stimulation intensity required to elicit eye twitch with multiple-channel field stimulation compared to single-field stimulation. Moreover, complete eye closure was only observed in cases of multiple-channel stimulation [22].

To date, there is no study comparing the effects of different electrode application sites on lid closure when orbicularis oculi is stimulated in humans. Most studies in humans have either focused on stimulation of the peripheral nerve branch leading to the orbicularis oculi [11, 15] or direct stimulation of the orbicularis oculi muscle [10, 12–14], without making a comparison between the two approaches. When stimulation of the orbicularis oculi occurs by stimulation of the peripheral facial nerve, localizing the exact anatomical region of the nerve can be difficult.

If a future prosthetic device were to target the orbicularis oculi directly, it would be ideal to stimulate both the lower and the upper orbicularis oculi. The palpebral part of the orbicularis oculi in the lower lid plays a crucial role in the tear drainage system. For patients with a negative Bell phenomenon, stimulating the lower lid may be essential to minimize the risk of exposure to keratopathy.

In the design of such a prosthetic device, it should be considered that the palpebral part of the orbicularis oculi is shorter in the lower eyelid than in the upper eyelid, potentially requiring less electrical stimulation for sufficient contraction [34]. Additionally, the tarsus lying beneath the orbicularis oculi in both the upper and lower eyelids could serve as a protective shield for the eye against repetitive electrical stimulation. Multiple-channel stimulation may be beneficial to achieve muscle contraction with minimal stimulation intensity, as discussed above [22]. Studies are needed to assess the ideal placement sites for electrodes in humans and to investigate potential adverse effects.

## Blink detection of the healthy side in closed-loop electrical stimulation systems

For the development of an efficient closed-loop device capable of processing an output signal for stimulating the affected orbicularis oculi, an input signal must be acquired. As previously described, this signal is typically obtained from the healthy orbicularis oculi muscle. One common method for detecting orbicularis oculi activity from the healthy eye is electromyography (EMG). EMG registers muscle activation before movement, which is crucial for rapid signal processing and inducing muscle stimulation to the contralateral orbicularis oculi [14, 21, 23, 27, 28, 35]. However, the activity of other muscles, such as the frontalis, masseter, and zygomatic muscles involved in smiling or chewing, can cause interferences in the EMG signal of the orbicularis oculi muscle [26, 28]. This interference could lead to artificially induced synkinesis, resulting in unintended eye blinking during chewing or laughing [28].

To address this issue in their closed-loop system, Frigerio et al. developed a software that considers not only orbicularis oculi EMG, but also EMG signals from the zygomaticus and masseter muscles, thereby helping to reduce these unwanted triggers [11, 28]. Consequently, when zygomatic or masseter activity is present without corresponding orbicularis oculi activity, no stimulation signal output for the contralateral side is computed. Additionally, Frigerio et al. incorporated a resting interval after each stimulus [11, 28]. They found that placing electrodes for EMG of the orbicularis oculi in a supero-nasal position yielded better recording, than supero-temporal, infero-nasal, or infero-temporal electrode placements [35].

Marcelli et al. devised a compact gyroscope, exploiting the concept that eyelid movement during blinking resembles a rotation with an axis through the temporal and lateral canthus. They affixed this gyroscope to the upper eyelid, allowing for the quantification of even the slightest eyelid motion [10]. A closed-loop device built on this gyroscope method could serve as a valid alternative to EMG [10].

Further, infrared-equipped blink detection glasses were examined for muscle activity detection. However, the latency between blink detection and intended stimulation was deemed inadequate for achieving a synchronous eye blink [36]. Another approach for detecting muscle activity involves recording the extracellular neural action potential of the facial nerve. Despite its high precision, this method comes with the drawback of the nerve signals being 20 times smaller in amplitude compared to EMG signals from muscles, which can pose challenges for practical use [26].

## Addressing the different side effects

### Pain

Despite the impaired function of the orbicularis oculi muscle in facial nerve palsy, the sensory trigeminal innervation remains intact, and therefore, pain sensation can persist. It is well established that electrical stimuli can be perceived as painful by the recipient, with the perception of pain increasing with higher amplitude levels [12, 26]. Stimulation at frequencies lower than 100 Hz tends to be more painful than at frequencies above 100 Hz [10, 11]. Additionally, single-pulse stimulation has been described as more painful than pulse-train stimulation, which could be attributed to the lower stimulation intensity required to induce eye closure in pulse-train stimulation [10]. Pain perception can be reduced by optimizing stimulation settings for eliciting eye closure and applying the lowest possible stimulus intensity.

To date, reported pain levels for electrical orbicularis oculi stimulation on a pain scale have ranged between 3 (± 1.7 SD) and 4.3 (± 2.6 SD) on a scale of 1 (no pain) to 9 (severe pain) [12, 13]. These sensations have been more accurately described as discomfort rather than pain. In a study by Frigerio et al. patients reported a pain score between 2–4 on a scale of 0 (no pain) and 10 (severe pain) [15].

### Synkinesis

Physiotherapist commonly employ early muscle activation techniques to prevent synkinesis in patients with facial nerve palsy [37, 38]. However, concerns have been raised regarding whether electrical stimulation of the orbicularis oculi muscle may induce synkinesis, leading to involuntary contraction of the levator muscle [12]. There is one study that reported the absence of synkinetic movements following repeated electrical stimulation of the eyelid over a period of up to 3 months [39]. Nonetheless, no randomized controlled studies study the development of synkinesis after electrical stimulation; therefore, further studies are required.

### Further considerations of repetitive electrical orbicularis oculi stimulation

In the context of repetitive electrical stimulation, it is important to consider that this may not only induce muscle contraction but also potentially lead to vasoconstriction [40]. Additionally, strong electric fields have the potential to cause cell membrane damage or activate blood platelets, although Brinton et al. found no evidence of damage at low levels of 20 and 150 V [40]. They also noted that tissue heating due to electrical stimulation remained below 1 °C at the electrode site, making it unlikely to induce vasoconstriction [40]. However, it is important to note that this was studied for a short duration of less than 30 min [40].

Given the thin nature of the eyelid, it is necessary to assess whether repetitive electrical stimulation could result in direct eye damage, such as corneal burns or scarring leading to eyelid malposition. Furthermore, it should be explored whether adjusting the stimulation parameters during night time, possibly using a low-intensity stimulation, is necessary. The potential side effects of prolonged electrical stimulation of the orbicularis oculi muscle in humans have not been comprehensively assessed, underscoring the need for further studies.

## Which patients should be targeted for electrical stimulation?

Patients deemed suitable candidates for eyelid reanimation via electrical stimulation require an intact orbicularis oculi muscle. There is available data indicating that early initiation of electrical stimulation in denervated quadriceps muscles in humans results in more pronounced clinically relevant improvements in muscle function. Nevertheless, even patients with long-standing or complete quadriceps denervation have demonstrated some degree of functional improvement following electrical stimulation, albeit to a lesser extent [17]. However, it has not yet been established whether these findings are applicable to electrical stimulation of the orbicularis muscle, as no randomized controlled studies in humans have investigated the response to varying degrees of muscle denervation.

In animal models, higher electrical stimulation intensity has been reported in partially denervated orbicularis oculi muscles compared to those with normal innervation [19]. Among human subjects, there is a trend indicating that the orbicularis oculi muscle responds to electrical stimulation even in cases of severe denervation, although the response may be more pronounced in milder denervation as opposed to severe denervation [13]. Nonetheless, the same study found no response to electrical stimulation in completely denervated muscles [13].

The findings mentioned above may imply that patients with longstanding facial palsy may potentially benefit from electrical stimulation. However, given the possibility of a more favorable response of the orbicularis oculi muscle to electrical stimulation in cases of mild denervation compared to severe denervation [13], it is essential to investigate whether the early commencement of electrical stimulation following facial nerve palsy might be advantageous in the context of orbicularis oculi reanimation. Further studies are necessary to validate these observations and to examine the criteria for selecting patients for electrical stimulation of the orbicularis oculi muscle.

## Conclusion

**Fig. 1:**
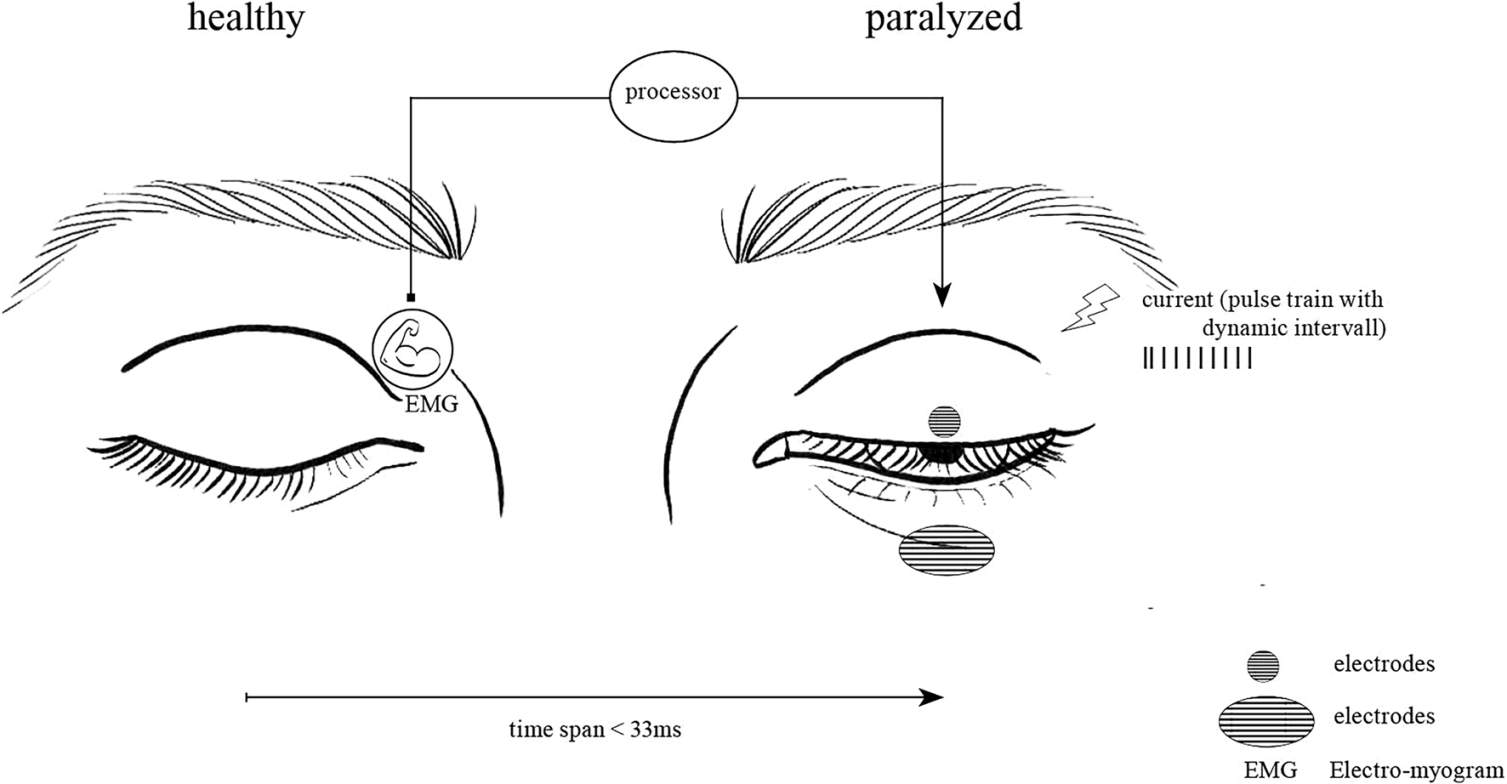
Visualization of a portable future closed-loop electrical stimulation device for patients with facial nerve palsy. Stimulus input signal can be recorded with EMG from the healthy side. Signal processing and electrical stimulation output generation should be processed in less than 33 ms. The output current should contain pulse train stimulation with a dynamic interval

Electrical stimulation of orbicularis oculi muscle is a promising tool in the treatment of facial nerve palsy already widely investigated in non-clinical papers. These studies show the feasibility of electrical stimulation in humans, showing the potential for restoring orbicularis oculi muscle function [10–15]. Presently, there is no readily available portable device designed for electrical stimulation of the orbicularis oculi muscle in humans. Such a portable device holds the potential to obviate the need for surgical intervention and enhance the quality of life for affected patients. The objective of this review is to provide a comprehensive overview of the current research as a foundation to move the knowledge from bench to bedside and develop a portable electrical stimulation device for the orbicularis oculi muscle for patients suffering from facial palsy (Fig. 1).

Patients with milder degrees of denervation may derive greater benefits from an electrical stimulation device than those with severe denervation [13]. However, there is a lack of randomized controlled studies delineating which patients are most suitable for electrical stimulation. An effective portable device should possess the capability to synchronize stimulation with the contralateral healthy side to achieve a natural-looking eyeblink [10, 11, 14, 23, 26–28, 35]. Synchronization could be accomplished through EMG measurements of the orbicularis oculi muscle, while accounting for potential interference from other facial muscles [11, 14, 26–28, 35]. The input signal from the healthy side must be swiftly processed to generate an immediate output stimulus for the affected orbicularis oculi muscle, with a latency of less than 33 ms, as this duration appears to be the threshold for human recognition of facial asymmetry [15, 30, 31].

Despite promising outcomes in current studies, additional research is imperative to establish safety parameters, determine optimal current settings and their effects, as well as assess potential side effects. The tolerability and refinement of ongoing electrical stimulation, employing the lowest feasible current, must be evaluated in human subjects. Further adjustments of stimulation settings are required to account for interindividual or intraindividual variations due to factors such as swelling, ischemia, atrophy, or variations during sleep, as well as the reduction of stimulation intensity in patients experiencing progressive facial nerve recovery. Subsequent research efforts are necessary to expand our understanding of electrode stimulation and define device specifications, including electrode placement, input and output signal processing, and the prevention of potential side effects associated with electrode stimulation therapy.
